# Assembly and use of high-density recombinant peptide chips for large-scale ligand screening is a practical alternative to synthetic peptide libraries

**DOI:** 10.1186/s12864-017-3814-3

**Published:** 2017-06-08

**Authors:** Harald Hundsberger, Kamil Önder, Peter Schuller-Götzburg, Dezso P. Virok, Julia Herzog, Raphaela Rid

**Affiliations:** 1 Department of Medical and Pharmaceutical Biotechnology, University of Applied Sciences, 3500 Krems, Austria; 2 Research Program for Rational Drug Design in Dermatology and Rheumatology, Department of Dermatology, Paracelsus Medical University of Salzburg, 5020 Salzburg, Austria; 3 ProComCure Biotech, 5081 Anif, Austria; 4 Research Program in Prosthetics, Biomechanics and Biomaterials, Paracelsus Private Medical University, 5020 Salzburg, Austria; 5 Department of Medicinal Microbiology and Immunobiology, University of Szeged, Szeged, 6722 Hungary

**Keywords:** Peptide library, Recombinational cloning, Recombinant peptide array, Protein chip, Peptide screening, Protein tags, Ligand design, High affinity, Target binding, Diversity

## Abstract

**Background:**

Recombinant peptide chips could constitute a versatile complementation to state-of-the-art *in situ* (chemical on-chip) synthesis, particle-based printing, or pre-manufactured peptide spotting. Bottlenecks still impeding a routine implementation - from restricted peptide lengths, low diversity and low array densities to high costs - could so be overcome.

**Methods:**

To assess overall performance, we assembled recombinant chips composed of 38,400 individual peptide spots on the area of a standard 96-well microtiter plate from comprehensive, highly diverse (>107 single clones) short random peptide libraries.

**Results:**

Screening of altogether 476,160 clones against Streptavidin uncovered 2 discrete new binders: a characteristic HPQ-motif containing VSHPQAPF and a cyclic CSGSYGSC peptide. Interactions were technically confirmed by fluorescence polarization as well as biolayer-interferometry, and their potential suitability as novel detection tags evaluated by detection of a peptide-fused exemplary test protein.

**Conclusion:**

From our data we conclude that the presented technical pipeline can reliably identify novel hits, useful as first-generation binders or templates for subsequent ligand design plus engineering.

## Background

Chips exposing precisely arranged spots of peptides on top of a solid support constitute a fairly young alternative to widely implemented display [1, 2] or protein-fragment complementation [3, 4] methods - each with its unique strengths and weaknesses - for extracting functional target binders from combinatorial peptide libraries [5, 6]. Their popularity is at least in part attributable to the rising appreciation of peptides as starting points for the design of novel therapeutics - ideally exhibiting favourable safety, tolerability plus efficacy profiles by merging advantages of traditional small molecule drugs (conformational constraints, membrane permeability, oral bioavailability, metabolic stability, lower fabrication complexity) with those of proteins (natural occurrence, high target specificity/selectivity) [7]. As highlighted elsewhere [8–11], peptide chips can in essence be assembled by 3 different procedures, namely parallel on-chip (photolithography, SPOT concept) synthesis, particle-based printing, or deposition of chemically pre-synthesized peptides. Fodor and co-workers were amongst the first to convert Merrifield solid phase peptide chemistry [12] to a chip format. They introduced a set of photolabile ‘caps’ that only upon selective laser illumination allowed for the liberation of the N-terminus of a growing peptide chain, in this way making it possible to exactly guide repetitive cycles of light-directed de-protection, coupling, and washing away of unreacted monomers [13]. In contrast, the conceptually simpler, much more popular SPOT approach - launched by Frank and colleagues [14, 15] in continuing the previous achievements of Geysen *et al.* [16] - delivers small volumes of pre-activated amino acid (AA) solutions on a porous planar support (classically functionalized cellulose filter paper). Absorbed droplets create individual reaction compartments for subsequent parallel combinatorial peptide synthesis by standard Fmoc (fluorenyl-methoxy-carbonyl) cleavage processes [11, 17]. Particle-mediated layer-by-layer ‘laser printing’ [18, 19], next, relies on 20 different toner matrices that encapsulate chemically activated, on-demand addressable building blocks instead of regular colour pigments. When the latter are deposited and heated up, all the solid microparticles at once melt, triggering the release plus instantaneous coupling of the hitherto matrix-arrested AA (with yields similar to standard Merrifield synthesis). Spotted peptide chips, lastly, make use of robotic microarrayers to (chemoselectively) immobilize (nanolitre) volumes of pre-synthesized (longer-chain) peptide solutions onto coherent substratum [20, 21]. Solely this procedure offers the option to integrate quality control in the manufacturing process, and might under certain circumstances - e.g. when multiple copies of the same chip are required - be more efficient as each molecules needs to be synthesized only once.

**Table 1:** Strengths and limitations of recombinant peptide chips

Strengths of recombinant peptide chips
• Peptide libraries are created via recombinational cloning (low frequency of background colonies; maintenance of pre-defined orientation/reading frame; high cloning efficiency). • Design ranges from entirely random peptide collections to customized content (soft randomization; scaffold-based). • Peptide lengths are not limited as is still the case for state-of-the-art chemical on-chip synthesis, particle-based printing or chemically pre-manufactured peptide spotting. • Growth, induction and lysis of library-transformed *E. coli* clones all takes place on a single nitrocellulose membrane. • Evaporation or merging of spots is not a major concern. • The technique is conceptually facile, robust, cost-effective, sensitive, and easily (up-)scalable; fast turnaround times. • Has many promising applications, e.g.: epitope mapping, alanine substitutions, replacement studies, truncation scans, positional/ scrambled peptide library screening, along with unbiased examinations without any *a priori* knowledge. • Limited throughput can be compensated by massive parallelization (applying e.g. elaborate pooling schemes). • Extracted hits are immediately available as clones. • Integration of controls for quality estimation, affinity assessment, and inter-blot normalization is possible. • ‘Cell-free’ nature increases the chance of confirming hits with usual methods (FP, SPRI, Western Blot, Co-IP, etc.).
Limitations of recombinant peptide chips
• At present reachable density (resolution) represents an only sparse sampling of the theoretically possible combinatorial random (nonamer/hexamer) peptide library diversity. • Number of peptides that can be screened in a single approach is several orders smaller than feasible in typical yeast two-hybrid (Y2H) or phage display settings. • Technical equipment for robotic clone picking, reagent dispensing and/or clone arraying (printing) might be needed (depending on the desired throughput rate). • Currently only evaluated for moderate and higher affinity (strong) binding strengths, not for weak interactors. • Incorporation of modified or non-proteinogenic (synthetic) segments during chip compilation is not possible.

**Fig. 1:**
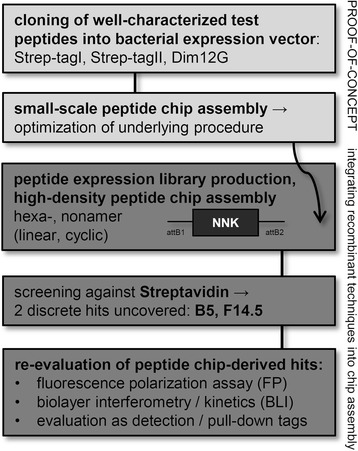
Experimental workflow

**Fig. 2:**
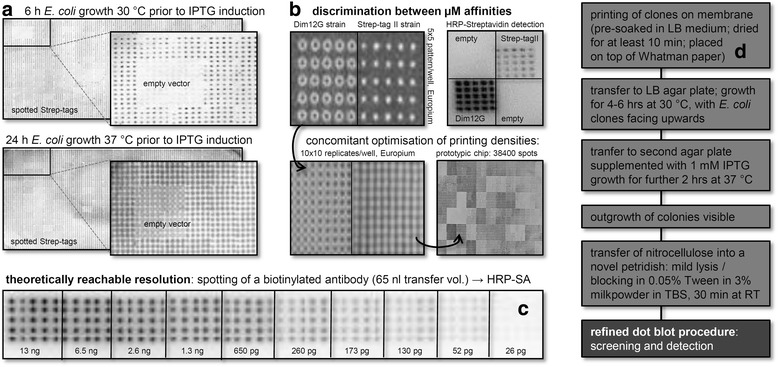
Recombinant peptide chip optimization. **a** Assessment of best *E. coli* growth intervals for consistent chip performance. A series of *E. coli* cells transformed with either Strep-tag constructs or empty vector were imprinted on nitrocellulose, grown thereon for the specified period, then exposed to IPTG, and subsequently lysed. Released peptides were analysed for their interaction with HRP-labeled Streptavidin. **b** Signal intensities (and so a rough estimation of affinities) between Strep-tagII and Dim12G were evaluated in relation to empty vector control, as indicated. Different peptide spot numbers per single standardized microtiter plate well are compared. **c** Consistent pin performance and maximal detection sensitivity were assessed by spotting a dilution series of biotinylated test protein. **d** Steps required for recombinant peptide chip assembly

Features like utmost aptitude towards miniaturization, multiplexing and automation equip this technology - irrespective of the underlying production method - with far-reaching applications, tentatively only restrained by combinatorial diversity plus realizable resolution. Incorporation of modified or non-proteinogenic (synthetic) segments during chip compilation furthermore offers access to a much wider chemical space. In practice, however, limited synthesizable peptide lengths as well as peptide numbers per area, inconsistent purities (accumulation of side products) and prohibitively high costs still impede a widespread adoption. The relatively small liquid droplets in SPOT synthesis, for instance, tend to either evaporate or merge with their neighbors, thus limiting manageable densities to 9 to maximally 25 peptides per cm^2^. Lithographic policies, in comparison, support much higher crowding, yet allow for only one type of AA building block to be sequentially connected, bringing about fairly low synthesis rates [19, 22, 23]. Even though first systemic ultra-dense arrangements (displaying 2.1 million overlapping 4-12mers) have only recently been pioneered [24, 25], recombinant peptide chips could definitely obliterate some of above-mentioned bottlenecks. To this end, we evaluated overall performance, practical diversity as well as throughput of recombinant peptide chips in a test screen against Streptavidin, and evaluated isolated hits with biochemical and biophysical methods. From our data we conclude that this conceptually simple, innovative technique could deliver further flexibility to basic and applied research. The underlying experimental workflow is summarized in Figs. 1 and 2d, and apparent strengths and limitations of this method are highlighted in table 1.

## Methods

### Subcloning of test constructs

Templates for Strep-tag I (5′-ggggacaagtttgtacaaaaaagcaggcttg-GCTTGGCGTCACCCGCAGTTCGGTGGT**TGA**tacccagctttcttgtacaaagtggtcccc-3′) [26], Strep-tag II (5′-ggggacaagtttgtacaaaaaagcaggcttgTGGAGCCACCCGCAGTTCGAAAAA-**TGA**tacccagctttcttgtacaaagtggtcccc-3′) [27] as well as a 12 glycine (G) linker spaced dimer (Dim12G) resembling a published Twin-conformation [28] (5′-ggggacaagtttgtacaaaaaagcaggcttg-TGGAGCCACCCGCAGTTCGAAAAA*ggtggtggtggtggtggtggtggtggtggtggtggt* GCTTGGCGTCACCCGCAGTTCGGTGGT **TGA**tacccagctttcttgtacaaagtggtcccc-3′) were *de novo* assembled by Eurofins MWG Operon as attB site-flanked oligonucleotides, and directly recombined into Gateway® entry vector pDONR™-zeo (life technologies™, Thermo Fisher Scientific). Resultant constructs were shuttled into bacterial expression vector pDEST™15 (glutathione S-transferase GST fusion plasmid, Thermo Fisher Scientific). Inserts were sequence-verified (ABI PRISM™ Big Dye Terminator Cycle Sequencing Ready Reaction Kit, Applied Biosystems).

### Acquisition of recombinant Streptavidin/StrepTactin

Recombinant Streptavidin and/or StrepTactin proteins, in either unconjugated format (for fluorescence polarization assays) or with horseradish peroxidase (HRP) or Europium labels, were purchased from Thermo Fisher Scientific, BioRad or Perkin Elmer, respectively.

### Peptide library construction

Templates for linear (5′-aaaaagcaggcttg[NNK]_6/9_ **TAA**tacccagctttct-3′) or cyclically constrained (5′-aaaaagcaggcttgTGT[NNK]_6/9_TGT**TAA**tacccagctttct-3′) hexamer or nonamer peptide libraries were ordered at Microsynth AG, and equipped on both ends with full recombinase recognition sites in the course of a standard PCR reaction (attB1-forward: 5′-ggggacaagtttgtacaaaaaagcaggcttg-3′; attB2-reverse: 5′-ggggaccactttgtacaagaaagctgggta-3′; denaturation at 94 °C for 30 s, annealing at 55 °C for 30 s, extension at 72 °C for 30 s; 40 cycles). Resultant PCR products were directly inserted into pDONR™-zeo by BP recombination. Two 10 μl reactions each containing equimolar ratios of 150 ng insert (peptide) DNA, 150 ng vector and 2 μl BP clonase™ II mix in 1× TE (Tris-EDTA, pH 8.0) were incubated at room temperature (RT, 25 °C) for 20 h. The mixture was then pooled, purified via Illustra’s GFX™ PCR DNA and Gel Band Purification Kit (GE Healthcare), transformed into 2 aliquots of electrocompetent Top10 *E. coli* cells (theoretical efficiency: 10^9^ cfu/μg supercoiled DNA, Thermo Fisher Scientific) and propagated on large (145 mm diameter, Nunc) low salt LB agar plates supplemented with 25 μg/ml Zeocin™ (Thermo Fisher Scientific). Serial dilutions were in parallel plated to assess the number of independent transformants containing unique inserts. The next day, clones were scraped, pooled, and plasmid DNA of resultant primary entry libraries prepared using the GenElute™ HP Plasmid Maxiprep kit (Sigma Aldrich). Part of the *E. coli* libraries was 1:1 supplemented with 87% glycerol and frozen at −80 °C for long-term storage. For final generation of screening-ready [NNK]_6/9_ or C-[NNK]_6/9_-C destination collections, respective constructs were shuttled into vector pDEST™15 using Gateway® LR clonase™ II Plus mix (Thermo Fisher Scientific) according to the manufacturer’s protocols, except that again a 5× scale was used and recommended proteinase K digestion was omitted [29]. Reactions were purified by running through Illustra’s spin columns (GE Healthcare), and transformed into 100 μl electrocompetent Top10 *E. coli* cells. For selection of successfully recombined vectors, cells were plated on 10 large LB agar supplemented with 100 μg/ml ampicillin. Transformants of two plates each were scraped from solid-surface agar, and plasmid DNA recovered. Insert diversity was estimated by sequencing 30 randomly picked clones each with vector-specific primers (pDEST15-fw = 5′-TAATACGACTCACTATAGGG-3′, T7P-rv = 5′-TAGTTATTGCTCAG-CGGTGG-3′).

### Recombinant peptide chip assembling and screening

Competent *E. coli* One Shot® BL21 Star™ (DE3) cells (Thermo Fisher Scientific) carrying the gene for T7 RNA polymerase under control of isopropyl β-D-1-thiogalactopyranoside (IPTG)-inducible *lac*UV5 promotor were transformed with respective expression libraries, and plated onto large square Q-trays. Individual colonies were robotically picked (QPix2^xt^, Genetix) into suitable 384 well plates containing liquid LB medium supplemented with 100 μg/ml ampicillin, and cultivated overnight at 37 °C. Alternative route was to directly split appropriately diluted (as confirmed by plating onto LB-amp agar plates) transformation reactions (after 1 h recovery in antibiotics-free SOC medium) into 384 well plates via a programmable Flexdrop Precision Reagent Dispenser (Perkin Elmer). The next day, clones were robotically arrayed onto a 0.45 μm pore size PROTRAN™ nitrocellulose sheet (Whatman) cut in the form of the original masterplate, applying a 1 × 1, 5 × 1, 5 × 5, or 10 × 10 pattern (such that each clone or pool is gridded in 1, 5 or 25 or 100 replicates each). Following a 6 to 8 h incubation interval on top of solid LB at 30 °C, peptide expression was achieved by transferring these membrane-imprinted clones onto LB agar plates supplemented with 1 mM IPTG, followed by a 2 h incubation interval at 37 °C. Cells were then directly lysed via transfer into a Tris-buffered saline (TBS) solution containing 0.05% Tween-20 and 3% blotting-grade milk powder. Membranes were in refined dot-blot reminiscent procedure challenged with (horseradish peroxidase) HRP- or Europium labeled Streptavidin (in 1:1000 to 1:5000 dilution in TBS-0.05% Tween) for 1 h at RT, washed 3 times in TBS-0.05% Tween, and scanned with either a ChemiDoc imaging system (Quantity One® analysis software, BioRad) after exposure to Immobilon™ Western chemiluminescent substrate (Millipore, Merck), or directly with a prototypic SpectraMax® Paradigm® multi-mode detection Platform (ScanLater™ Western Blot Detection Cartridge, Molecular Devices).

### Fluorescence polarization (FP)

FP assays were performed in ES2 buffer (100 mM potassium phosphate pH 7.4, 100 μg/ml bovine γ globulin, 0.02% NaN_3_), applying 80 μl reaction volumes per black-bottomed 384 microtitre plate well (Nalge Nunc). 20 nM (corresponding to circa 20,000 fluorescence counts) of 5’FAM-labeled Strep-tagI (synthesized by GenScript Inc.), Strep-tag II or any novel hit were incubated with rising concentrations of Streptavidin/StrepTactin protein (in triplicates each) for at least 2 h at RT (or preferably o/n at 4 °C) to ensure binding equilibrium. Fluorescence emission (535 nm) intensities from both parallel and perpendicular orientations were after excitation at 480 nm measured in a DTX Multimode Detector (Beckman Coulter Genomics), checked for absence of abnormal intensity changes (that might result from solvent interference), and polarization values (expressed as millipolarization mP units) calculated via the formula mP = 1000(*I*_s_-*GI*_p_)/(*I*_s_ + *GI*_p_), where *G* represents the *G f*actor (= *I*_s_/*I*_p_, settled as 0.650 for this study). Data were analyzed by Graphpad Prism 5 software, and the dissociation constant K_d_ calculated by non-linear regression (sigmoidal dose-response curve fitting).

### Bio-layer interferometry (BLI) binding kinetics

Binding between Streptavidin and Strep-TagII, B5 or F14.5 were in relation to a negative control peptide (5’FAM-CLNSVAGGG) analyzed on ForteBio’s BLItz® system. After recording of the primary baseline for 30 s in ES2 buffer, hydrated Streptavidin-coated biosensors were loaded with chemically synthesized peptides, and a potential increase in BLI signal measured for 2 min. Dissociation was assessed by transferring the loaded biosensor to a tube containing buffer only. Affinities (K_d_) were calculated using the embedded BLItzPro 1.2 software.

### Fusion of chip-derived hits B5 and F14.5

Templates for 12G–spaced dimeric F14.5 (5′-aaaaagcaggcttgTGTTCGGGGAGTTAT-GGGTCGTGTggtggtggtggtggtggtggtggtggtggtggtggtTGTTCGGGGAGTTATGGGTCGTGT**TAA**tacccagctttct-3′) and in analogous fashion for B5 were ordered at Microsynth AG and amplified with attB primers. After subcloning in pDONR™-zeo and further in pDEST™15, any observable change in interaction strength was evaluated on recombinant peptide chip level in relation to enclosed Strep-tagII and Dim12G control constructs.

### Recombinant protein production and Western Blot analysis of B5/F14.5 tagged *S. aureus* ClfB

Primers for C-terminally equipping an exemplary 38 kDa *S. aureus* ClfB fragment (SAV2630, NP_373154, [30]) with Strep-tag I (Clfb-fw = aaaaagcaggcttgAGTTTAGCTGTTGCTGAACCGG; rv = 5′-gtacaagaaagctgggta**TTA**accaccgaactgcgggtgacgccaagcATTTACTGCTGAATCACCATCAGC-3′, Strep-tag II (rv = 5′-tacaagaaagctgggta**TTA**tttttcgaactgcgggtggctccaATTTACTGCTGAATCACCATCAGC-3′, F14.5 (rv = 5′- gtacaagaaagctgggta**TTA**acacgacccataactccccgaacaATTTACTGCTGAATCACCATCAGC-3′ and B5 (rv = 5′-GTACAAGAAAGCTGGGTA**TTA**gaaaggggcctgcggatgagaaacATTTACTGCTGAATCACCATCAGC-3′ were designed. After BP recombination of resultant PCR-products in pDONR™-zeo, inserts were recombined in pDEST™17 (attaching an additional N-terminal 6xHIS-tag). For small-scale purification, ClfB from soluble, IPTG induced supernatants was captured on pre-equilibrated magnetic Streptavidin coated Dynabeads**®** (Thermo Scientific) in PBS on a rotary shaker, washed 4 times in PBS containing 0.1% bovine serum albumin, eluted by harsh boiling for 2 min in 1× SDS sample buffer, and subjected to 12% SDS-PAGE electrophoresis. Following transfer to nitrocellulose and blocking in 3% non-fat milk in TBS-0.05% Tween for 1 h at RT, membranes were probed either directly with HRP-Streptavidin / StrepTactin (1:1000) or with an α-6xHIS HIS (SantaCruz) / HRP-α-mouse antibody (1:10,000, Agilent Technologies) combination. For dot blot analysis, 1 μl of total cell lysates, soluble supernatants, and recovered eluates were manually spotted each.

## Results

### Both novel and cognate Streptavidin binders can be extracted from recombinant peptide chips

Screening parameters were first of all optimized: signal-to-background ratios and realizable detection limits were assessed by analyzing the associations between published Strep-tagI and II peptides (as well as 12G linker spaced dimer derived thereof) and either commercially available HRP- or fluorescence labeled Streptavidin. Our preliminary screening of a recombinant test peptide chip content assembled from respective control strains (Fig. 2) permitted us to clearly distinguish both moderate (Strep-tagI and II) and higher affinity (Dim12G) binders in comparison to the enclosed negative control, pointing to the overall practical feasibility of such an approach. We next further optimized *E. coli* growth times (from 0 to 24 h at 2 different temperatures), induction intervals, critical cell densities required, printing conditions, lysis and detection conditions, etc. (Fig. 2). Incubation of the imprinted membranes for 6 to 8 h at 30 °C on top of LB-amp plates prior to IPTG induction - but in no case overnight at 37 C because of excessively accumulating bacterial biomass - yielded acceptable results for Strep-tag constructs in comparison to empty vector reference (for both HRP and Europium detection). Despite leaky basal expression and hence observable differences even at time point 0 (lysis of clones directly after arraying), IPTG induction definitively triggered increased peptide expression levels, thereby delivering more consistent results. Our applied low concentration of 0.05% Tween-20 as weak non-ionic detergent lysed enough bacterial cells to release sufficient recombinant material for immediate immobilization, but simultaneously maintained the ability of fluorescent control proteins to emit light (data not shown), suggesting that it does not denature peptides/proteins but instead preserves their native (−like) conformation. Strep-tagI, Strep-tagII and Dim12G were further exploited for fine-tuning printing densities (5 × 5 pattern for 25 replicates, to at present maximally 100 spots on the area of a single 384 microtiter plate well), and were enclosed as internal controls in subsequent chip assembly efforts for standardization, quality estimation, affinity assessment, as well as inter-blot normalisation.

**Fig. 3:**
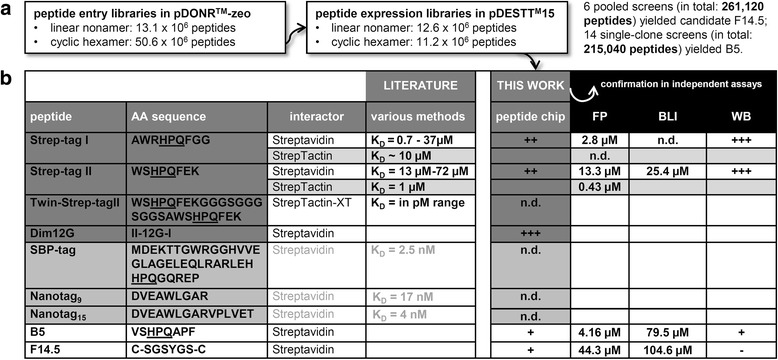
Performance of literature-validated Strep-tags in our recombinant peptide chip setup, and comparison of target binding affinities to two novel chip-derived binders. **a** Nature and diversity of random peptide expression libraries applied for this study. **b** Detailed comparison of both known (see discussion section) and novel Streptavidin/StrepTactin binders. SBP and Nanotags (highlighted in light grey) have not been included in the present study. WB, Western Blot

*Bona fide* performance in large-scale discovery screening was in a next step addressed by assembling random peptide chips from comprehensive random recombinant peptide libraries, all prepared by in-house up-scaled recombinational cloning procedures [29]. These libraries were designed to carry either a linear or a cyclically constrained degenerate NNK triplet configuration (where N denotes an equimolar 25% mixture of all 4 deoxynucleotides; and K represents a 50% balanced mix of each thymine and guanine), thereby incorporating all 20 natural AA (redundant codon reduction from 64 to 32) yet merely one single amber TAG stop codon [31]. Transformation efficiencies, overall insert diversities, and lack of apparent bias were determined for all independently cloned entry and destination resources (Fig. 3a) by counting the number of *E. coli* clones grown on antibiotics-supplemented LB agar plates, followed by sequencing 100 randomly picked single *E. coli* clones each. Following the above-established workflow, 2 hits (retrieved from the corresponding position in the original microtiter masterplate; identification via sequencing of isolated plasmid DNA) could be selected.

**Fig. 4:**
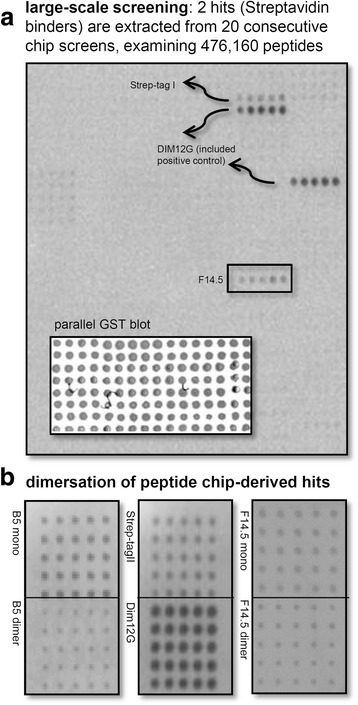
Uncovery of novel Streptavidin binders by recombinant peptide chips. **a** Excerpt of a random peptide chip (5 technical replicates each), resulting in the discovery of F14.5. Positive controls were included for simultaneous semi-quantitative evaluation of binding strengths. An in parallel performed GST-blot reveals relatively homogenous peptide expression levels. **b** Dimerisation of B5 and F14.5, analyzed by recombinant peptide chip

Candidate B5 (VSHPQAPF, premature stop) was uncovered from altogether screened 215,040 random peptides arbitrarily selected from 12.6 × 10^6^ clones of linear nonamers. F14.5 (C-SGSYGS-C), on the other hand, was extracted from 261,120 spotted random cyclic hexamers (11.2 × 10^6^ combinations) by directly dispensing diluted transformation mixes and re-analysing positive pools on single-clone level. The success rate was therefore 2 binders from 476,160 screened recombinant peptides. Since Dim12G, a fusion of Strep-tagI and Strep-tagII monomers, performs superior in comparison to a single monomer, our identified candidates B5 and F14.5 were subsequently dimerized (spaced by an analogous 12 glycine linker), and investigated for increased affinity (Fig. 4b). Whilst the HRP-Streptavidin mediated signals remained fairly constant for both F14.5 mono- and dimer, B5 monomer functioned slightly better than its merged counterpart. In both cases we could not increase affinity by linear dimerization, indicating that our hits rather function in a monomeric state.

### Chip-derived hits can be confirmed in independent assays, underscoring target binding specificity

**Fig. 5:**
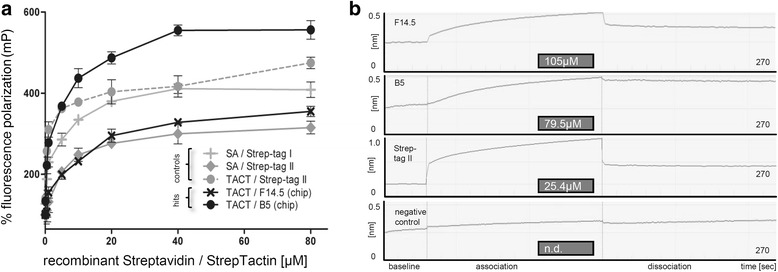
Re-evaluation of chip-derived Streptavidin binders in complementary setups. **a** *In vitro* fluorescence polarization assays using constant amounts of 5’FAM labeled (chemically synthesized) peptides plus increasing concentrations of recombinant protein. Data are reported as mean from 3 independently performed measurements. Standard deviation was below 15% for all values. **b** Binding kinetics (association of pre-determined amounts 5’FAM labeled peptides to Streptavidin biosensors) were measured via biolayer-interferometry. K_d_ values as calculated from the BLItz Pro software are listed

We confirmed chip-derived hits B5 and F14.5 in cell-free assays (see Fig. 5), ruling out false-positives (unspecific ‘sticky’ binders). An FP assay - conceptually based on rotational differences between labeled peptides free in solution versus complexed to an interactor - was arranged for chemically synthesized 5’FAM-labeled peptides Strep-tagI, Strep-tagII, B5, and F14.5. We monitored expected dose-dependent increases in polarization with increasing amounts of recombinant protein for Strep-tagI (K_d_ = 2.75 μM for Streptavidin) and StrepTag II (K_d_ of 13.26 μM for Streptavidin; literature-conform superior K_d_ of 0.43 μM for StrepTactin). Saturation binding could also be observed for B5 (K_d_ = 4.16 μM for StrepTactin) as well as F14.5 (K_d_ = 44.29 μM). Despite differences in slopes and amplitudes, both interaction potential and target specificity were in this way evaluated. Secondly, label-free biolayer interferometry (BLI) was measured on a disposable Streptavidin-coated biosensor which we exposed to respective peptides, thereby leading to an increase in optical thickness at the tip and, simultaneously, a real-time measurable wavelength shift (interference pattern of the reflected light) proportional to binding. The calculated K_d_ values were 25.4 μM for the Strep-tagII positive control (initially very fast binding; to a lesser extent also observed in other buffers), 105 μM for F14.5, and 79.5 μM for B5. Although peptide solvent, applied assay buffer and the fact that only a single analyte concentration was tested admittedly influence detected outcomes, achieved results appear consistent with above data.

### Further engineering efforts might transform extracted hits into suitable binders

**Fig. 6:**
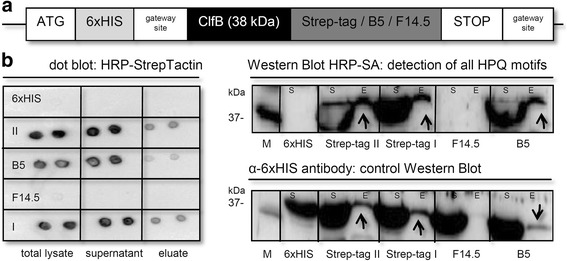
Functional analysis of hit peptides as putative affinity/purification reagents. **a** Schematic illustration of underlying cloning procedure. **b** *S. aureus* clumping factor was C-terminally tagged with either Strep-tag I, Strep-tag II, F14.5 or B5, as indicated, and shuttled together with the original (unmodified) construct in pDEST™17. Recombinant protein expression (total lysate; S: soluble supernatant) and subsequent ClfB (37 kDa) protein purification on magnetic Streptavidin beads (E: eluate) were detected on dot blot as well as Western Blot level (12.5% SDS-PAGE), applying either HRP-labeled Streptavidin/StrepTactin or an α-6xHIS control antibody. Molecular mass standards (M) are indicated on the left

In order to evaluate whether B5 or F14.5 might be directly used as novel Western Blot detection or even as affinity purification agents, we equipped an exemplary 6xHIS-tagged test protein (*S. aureus* clumping factor ClfB; chosen because it can be produced in soluble form and acceptable yield) with respective peptides (Fig. 6). Comparable signals were observed on both dot-blot (HRP-StrepTactin) and Western Blot (HRP-Streptavidin) level for Strep-tagI and Strep-tagII in crude lysed cell extracts or soluble supernatants (obtained thereof after centrifugation clearance). HPQ-containing B5 could be successfully detected as well, albeit at reduced intensity. We next tried to affinity-purify all 4 tagged proteins (in relation to unmodified ClfB, carrying solely a 6xHIS-tag) by magnetic Streptavidin beads under physiological buffer conditions. Consistent with above results, B5 performed acceptably (yet poorer than Strep-tagsI and II), whilst F14.5-tagged ClfB could only be detected in an α-6xHIS antibody control blot.

## Discussion

Recombinant peptide chips take advantage of directly growing, inducing and lysing library-transformed *E. coli* clones on a single nitrocellulose membrane for subsequent screening with labelled proteins or compounds, thereby constituting a HTP-compatible alternative to presently available synthetic chemistry setups. We have previously already demonstrated the theoretical feasibility of proposed tactic by mapping the roughly specified epitope of a commercial antibody against vitamin D3 receptor by screening 2304 overlapping peptides to a 27 AA encompassing continuous stretch [32]. Many methodical aspects have since then been fine-tuned (Fig. 2), amongst them in-depth evaluation of most optimal *E. coli* growth and IPTG induction times/temperatures, printing densities (25 to maximal 100 spots per peptide), etc. In continuation, the present work now examines recombinant peptide chips under optimized conditions (e.g., signal-to-background ratios, interactor affinity ranges, practically reachable density) by basically exploiting the well-described interactions between Streptavidin and short artificial Strep-tag I (AWRHPQFGG, published K_Ds_ of 0.7–37 μM, strongly dependent on accomplished assay type), Strep-tag II (WSHPQFEK, K_D_ of 13–72 μM) as well as a 12G–linker spaced dimer as internal controls and reference (summarized in Fig. 3). Strep-tag I specifically binds to a proteolytically truncated ‘core’ version of Streptavidin by occupying the same pocket where biotin as the natural ligand with a dissociation constant around 10^−^15 M normally gets non-covalently complexed [26, 33, 34]. It was isolated from a genetic library as novel affinity reagent for the purification of fusion proteins on Streptavidin matrices at high purity and maintained functionality/bioactivity. Systematic optimizations have over the years yielded Strep-tag II (and subsequent introduction of a dimeric ‘twin’ tag) which exhibits not only higher intrinsic affinity towards StrepTactin, but also permits greater flexibility in choice of attachment site (N- as well as C-terminal fusion) [28, 34]. Further parallel *in vitro* selection attempts have parenthetically brought about the 38 AA encompassing SBP-tag with an equilibrium dissociation constant of 2.5 nM [35, 36], and the 9mer (17 nM) or 15mer (4 nM) Nano-tags with their nanomolar affinities [37].

We screened Streptavidin (mainly chosen because it shows - as widely applied purification and immunoassay detection reagent - low unspecific binding properties, and has been successfully applied in several previous phage and mRNA display selections [38]) against our recombinant peptide chips. The latter were built from either linear or disulfide-bond constrained cyclic libraries. Respective peptide populations (Fig. 3a) consisted on average of 6 × 10^6^ to more than 1 × 10^7^ diverse single clones (not taking into account point mutations, frameshifts or premature stops that might be caused by proofreading activity-lacking Taq polymerase and the applied PCR conditions, thereby further enrichening the repertoire). Despite this substantial combinatorial diversity (which again is proportional to the probability of uncovering peptides with desired properties [39]), this number still represents an only sparse sampling of the theoretically possible combinatorial library diversity. The theoretical sequence space is up to 20^6^ or 20^9^ inserts, for an exhaustive hexamer or nonamer library, respectively. At present, a maximum of 38,400 spots can be displayed on a standard microtiter-plate sized membrane (under optimized conditions, with pin based usual DNA/protein spotters). This number is several orders smaller than feasible in typical Y2H or phage display screening sceneries, and represents only a tiny fraction of the theoretical possible diversity of a nonamer/hexamer library. This challenge was compensated by parallelization, i.e., by performing either many simultaneous screens, or by initiating elaborate pooling schemes (dispensing of appropriately diluted transformation mixes) that initially test pools, e.g. 10 clones per array position first (which increases the throughput 10 times) and subsequently re-analyse positive pools on single-insert level. In doing so, 2 Streptavidin binders could be successfully uncovered, i.e., candidate B5 from a representative number of 215,040 linear nonamers, and F14.5 from 261,120 tested cyclically constrained hexamers. This number is still far below cell-based screening techniques, however completely cell-free and based on a biochemical principle, which increases the chance that first hits are confirmed with usually used confirmation methods (FP, SPRI, WB, Co-IP, etc.). Practically considered, a number of ~500 k different peptides represents an impressive and useful diversity for screening approaches. Candidate B5, of note, contains the typical HPQ core consensus motif characteristic for biotin mimetics, underscoring that recombinant peptide chips built from such random libraries indeed constitute a source for specific, functional target binders [40]. The lower affinity compared to the original Strep-tags for both Streptavidin and StrepTactin can be explained by the flanking AA residues that alter the conformation of the central HPQ tripeptide. F14.5 with its attached cysteine residues, in contrast, does not contain any known pattern [38, 41], and can hence be considered as a new peptide with a different binding mechanism.

The specificities of B5 and F14.5 were finally re-evaluated in complementary experimental setups. For technical confirmation, we used fluorescence polarization and bio-layer interferometry, which delivered also affinity data. A functional evaluation was based on recombinant expression and affinity purification of a tagged test protein. The measured dissociation constants of B5 and F14.5 (4.16 μM and 44.29 μM for StrepTactin in FP studies versus 79.5 μM and 104.6 μM in bio-layer interferometry kinetic analyses) further corroborated target binding specificities, albeit at significantly lower affinities compared to the enclosed Strep-tag positive controls. B5 (attached to ClfB test protein) could on Western Blot level be successfully detected in crude bacterial cell lysates, and proved even functional in affinity purification of ClfB on Streptavidin beads. These latter 2 applications were, in contrast, not feasible with F14.5, indicating most likely sterical or conformational problems of this tag in fusion to the test protein, calling for further engineering/maturation efforts (inclusion of a linker, analysis of other test proteins etc.).

## Conclusion

Our recombinant peptide chip technology couples the merits of recombinant protein technologies and combinatorial cloning strategies with high-density peptide chip assembly and high-throughput screening (see table 1). Features like conceptual straightforwardness, robustness and cost-effectiveness along with sensitivity, consistency as well as fast turnaround times equip this scalable, easily upscaling feasible setup with promising applications. Examples range from epitope mapping (wherein a protein under study is meticulously split into biologically active fragments) to systematic alanine substitutions, replacement studies (for investigating the contribution of distinct AAs and their possible exchanges), truncation scans (highlighting the minimum essential length of a functional peptide) and finally positional or scrambled (carrying permutations on the original sequence) peptide library screenings. Unbiased examinations of comprehensive peptide collections without any *a priori* knowledge, or, of an entire proteome translated into sets of overlapping peptides might yield unexpected associations missed in limited throughput. On the other hand, customized content (soft randomisation of first-generation hits by introducing degeneracy via wobble codon mixtures; scaffold-based design) can be launched without any size constraints, enabling a further maturation of interaction strengths or target specificities. Peptides uncovered by high-density recombinant peptide chips might therefore offer new possibilities for diagnostic, therapeutic, and basic research purposes. In summary, we could here present the construction and practical application of recombinant peptide chips in a large scale approach which is neither technically nor economically feasible by synthetical peptide strategies.

## Abbreviations

AA: amino acid
GST: glutathione S-transferase
HRP: horseradish peroxidase
IPTG: isopropyl β-d-1-thiogalactopyranoside
K_d_: dissociation constant
RT: room temperature

## References

[1] Wu CH (2016). Advancement and applications of peptide phage display technology in biomedical science. J Biomed Sci.

[2] Wada A (2013). Development of Next-Generation Peptide Binders Using In vitro Display Technologies and Their Potential Applications. Front Immunol.

[3] Yang M, Wu Z, Fields S (1995). Protein-peptide interactions analyzed with the yeast two-hybrid system. Nucleic Acids Res.

[4] Rid R (2010). “Renaissance” of the Yeast Two-Hybrid System - Enhanced for Automation and High-Throughput to Support Proteome-Wide Research. Letters in Drug Design and Discovery.

[5] Liu BA, Engelmann BW, Nash PD (2012). High-throughput analysis of peptide-binding modules. Proteomics.

[6] Blikstad C, Ivarsson Y (2015). High-throughput methods for identification of protein-protein interactions involving short linear motifs. Cell Commun Signal.

[7] Fosgerau K, Hoffmann T (2015). Peptide therapeutics: current status and future directions. Drug Discov Today.

[8] Min DH, Mrksich M (2004). Peptide arrays: towards routine implementation. Curr Opin Chem Biol.

[9] Shin DS (2005). Combinatorial solid phase peptide synthesis and bioassays. J Biochem Mol Biol.

[10] Pellois JP (2002). Individually addressable parallel peptide synthesis on microchips. Nat Biotechnol.

[11] Hilpert K, Winkler DF, Hancock RE (2007). Peptide arrays on cellulose support: SPOT synthesis, a time and cost efficient method for synthesis of large numbers of peptides in a parallel and addressable fashion. Nat Protoc.

[12] Merrifield RB (1965). Solid-Phase Peptide Syntheses. Endeavour.

[13] Fodor SP (1991). Light-directed, spatially addressable parallel chemical synthesis. Science.

[14] Frank R (1996). and H. Overwin, *SPOT synthesis. Epitope analysis with arrays of synthetic peptides prepared on cellulose membranes*. Methods Mol Biol.

[15] Frank R (2002). The SPOT-synthesis technique. Synthetic peptide arrays on membrane supports--principles and applications. J Immunol Methods.

[16] Geysen HM, Meloen RH, Barteling SJ (1984). Use of peptide synthesis to probe viral antigens for epitopes to a resolution of a single amino acid. Proc Natl Acad Sci U S A.

[17] Fields GB, Noble RL (1990). Solid phase peptide synthesis utilizing 9-fluorenylmethoxycarbonyl amino acids. Int J Pept Protein Res.

[18] Stadler V (2008). Combinatorial synthesis of peptide arrays with a laser printer. Angew Chem Int Ed Engl.

[19] Loeffler FF (2016). High-flexibility combinatorial peptide synthesis with laser-based transfer of monomers in solid matrix material. Nat Commun.

[20] MacBeath G, Schreiber SL (2000). Printing proteins as microarrays for high-throughput function determination. Science.

[21] Tapia V (2007). Affinity profiling using the peptide microarray technology: a case study. Anal Biochem.

[22] Panicker RC, Huang X, Yao SQ (2004). Recent advances in peptide-based microarray technologies. Comb Chem High Throughput Screen.

[23] Volkmer R (2009). Synthesis and application of peptide arrays: quo vadis SPOT technology. Chembiochem.

[24] Forsstrom B (2014). Proteome-wide epitope mapping of antibodies using ultra-dense peptide arrays. Mol Cell Proteomics.

[25] Buus S (2012). High-resolution mapping of linear antibody epitopes using ultrahigh-density peptide microarrays. Mol Cell Proteomics.

[26] Schmidt TG (1996). Molecular interaction between the Strep-tag affinity peptide and its cognate target, streptavidin. J Mol Biol.

[27] Voss S, Skerra A (1997). Mutagenesis of a flexible loop in streptavidin leads to higher affinity for the Strep-tag II peptide and improved performance in recombinant protein purification. Protein Eng.

[28] Schmidt TG (2013). Development of the Twin-Strep-tag(R) and its application for purification of recombinant proteins from cell culture supernatants. Protein Expr Purif.

[29] Rid R (2013). From the ORFeome concept to highly comprehensive, full-genome screening libraries. Assay Drug Dev Technol.

[30] Haim M (2010). Cytokeratin 8 interacts with clumping factor B: a new possible virulence factor target. Microbiology.

[31] Mena MA, Daugherty PS (2005). Automated design of degenerate codon libraries. Protein Eng Des Sel.

[32] Maier RH (2010). Epitope mapping of antibodies using a cell array-based polypeptide library. J Biomol Screen.

[33] Skerra A, Schmidt TG (1999). Applications of a peptide ligand for streptavidin: the Strep-tag. Biomol Eng.

[34] Korndorfer IP, Skerra A (2002). Improved affinity of engineered streptavidin for the Strep-tag II peptide is due to a fixed open conformation of the lid-like loop at the binding site. Protein Sci.

[35] Fogen D (2015). Engineering Streptavidin and a Streptavidin-Binding Peptide with Infinite Binding Affinity and Reversible Binding Capability: Purification of a Tagged Recombinant Protein to High Purity via Affinity-Driven Thiol Coupling. PLoS One.

[36] Keefe AD (2001). One-step purification of recombinant proteins using a nanomolar-affinity streptavidin-binding peptide, the SBP-Tag. Protein Expr Purif.

[37] Lamla T, Erdmann VA (2004). The Nano-tag, a streptavidin-binding peptide for the purification and detection of recombinant proteins. Protein Expr Purif.

[38] Ostergaard S (1995). Novel avidin and streptavidin binding sequences found in synthetic peptide libraries. FEBS Lett.

[39] Noren KA, Noren CJ (2001). Construction of high-complexity combinatorial phage display peptide libraries. Methods.

[40] Devlin JJ, Panganiban LC, Devlin PE (1990). Random peptide libraries: a source of specific protein binding molecules. Science.

[41] Caparon MH (1996). Analysis of novel streptavidin-binding peptides, identified using a phage display library, shows that amino acids external to a perfectly conserved consensus sequence and to the presented peptides contribute to binding. Mol Divers.

